# Fast and effective mitochondrial delivery of ω-Rhodamine-B-polysulfobetaine-PEG copolymers

**DOI:** 10.1038/s41598-018-19598-2

**Published:** 2018-01-18

**Authors:** Nobuyuki Morimoto, Riho Takei, Masaru Wakamura, Yoshifumi Oishi, Masafumi Nakayama, Makoto Suzuki, Masaya Yamamoto, Françoise M. Winnik

**Affiliations:** 1 Department of Materials Processing, Graduate School of Engineering, Tohoku University, 6-6-02 Aramaki-aza Aoba, Aoba-ku, Sendai, 980-8579 Japan; 2 Frontier Research Institute for Interdisciplinary Sciences (FRIS), Tohoku University, Aramaki aza Aoba 6-3, Aoba-ku, Sendai, 980-8578 Japan; 3 Department of Chemistry, University of Montreal, CP6128 Succursale Center Ville, Montreal, QC H3C 3J7 Canada; 4 Department of Chemistry and Faculty of Pharmacy, University of Helsinki, FI-00014 Helsinki, Finland; 5 Center for Materials Nanoarchitectonics, NIMS, 1-1 Namiki, Tsukuba, Ibaraki, 305-0044 Japan

## Abstract

Mitochondrial targeting and entry, two crucial steps in fighting severe diseases resulting from mitochondria dysfunction, pose important challenges in current nanomedicine. Cell-penetrating peptides or targeting groups, such as Rhodamine-B (Rho), are known to localize in mitochondria, but little is known on how to enhance their effectiveness through structural properties of polymeric carriers. To address this issue, we prepared 8 copolymers of 3-dimethyl(methacryloyloxyethyl)ammonium propane sulfonate and poly(ethyleneglycol) methacrylate, p(DMAPS-ran-PEGMA) (molecular weight, 18.0 < *M*_*n*_ < 74.0 kg/mol) with two different endgroups. We labeled them with Rho groups attached along the chain or on one of the two endgroups (α or ω). From studies by flow cytometry and confocal fluorescence microscopy of the copolymers internalization in HeLa cells in the absence and presence of pharmacological inhibitors, we established that the polymers cross the cell membrane foremost by translocation and also by endocytosis, primarily clathrin-dependent endocytosis. The most effective mitochondrial entry was achieved by copolymers of *M*_*n*_ < 30.0 kg/mol, lightly grafted with PEG chains (< 5 mol %) labeled with Rho in the ω-position. Our findings may be generalized to the uptake and mitochondrial targeting of prodrugs and imaging agents with a similar polymeric scaffold.

## Introduction

Mitochondria, located within the cytoplasm of eukaryotic cells, are organelles devoted primarily to the generation of energy in the form of adenosine triphosphate (ATP) through oxidative phosphorylation. Disfunction of mitochondria due to genetic transformation of mitochondrial proteins or oxidative stress can result in severe diseases, including cancer, neurodegenerative conditions, metabolic disorders, Alzheimer’s disease, and diabetes^1–4^. Given the pivotal role of mitochondria aberrations in the emergence and progression of these diseases, much effort is devoted to the design of effective mitochondria-targeted therapies. The mitochondrion consists of several compartments that hinder the entry of foreign substances into its central part, the matrix. The outer membrane has pores formed by porin, a channel-forming protein. The pores are permeable to molecules of molar mass less than ~5,000 g/mol. The inner membrane is the site of eukaryotic cellular respiration, the major mechanism of ATP biosynthesis. It is highly invaginated creating numerous cristae that provide the large surface area necessary for ATP production. The morphology of the inner membrane, which is maintained by the mitochondrial contact site (MICOS) complex and the ATP synthase in the cristae, can be altered by the apoptotic factor tBID and by mitofilin^5^.

The most common strategy of mitochondrial delivery involves coupling a drug to small targeting molecules, peptides, or gene-encoding proteins. Lipophilic small molecules that carry a delocalized positive charge, such as triphenyl phosphonium (TPP) or Rhodamine (Rho) derivatives, are known to localize preferentially in mitochondria^6^. Their lipophilic nature and charge delocalization facilitate penetration through the cell membrane. After internalization they accumulate in the mitochondrion interior matrix, which is negatively charged. This process can be very fast. For instance, TPP is taken up by mitochondria within minutes, which justifies the well-established use of TPP-modified drugs for selective mitochondrial delivery^7–11^. Another strategy consists in loading a drug within nanocarriers decorated with mitochondrial targeting groups on their outer surface. This approach decouples the mitochondria-targeting function from the drug discovery process^12–16^. Cellular uptake of the nanocarriers usually occurs via endocytosis, followed by endosomal release and mitochondria delivery. Drug-loaded liposomes, decorated with TPP, rhodamine (Rho), or peptides containing mitochondria targeting sequences, have been used successfully as well^17–20^. Although fusogenic liposomes^21,22^ function well as mitochondrial delivery, they are not used extensively in view of their poor long term stability and complex formulation^23^.

**Figure 1:**
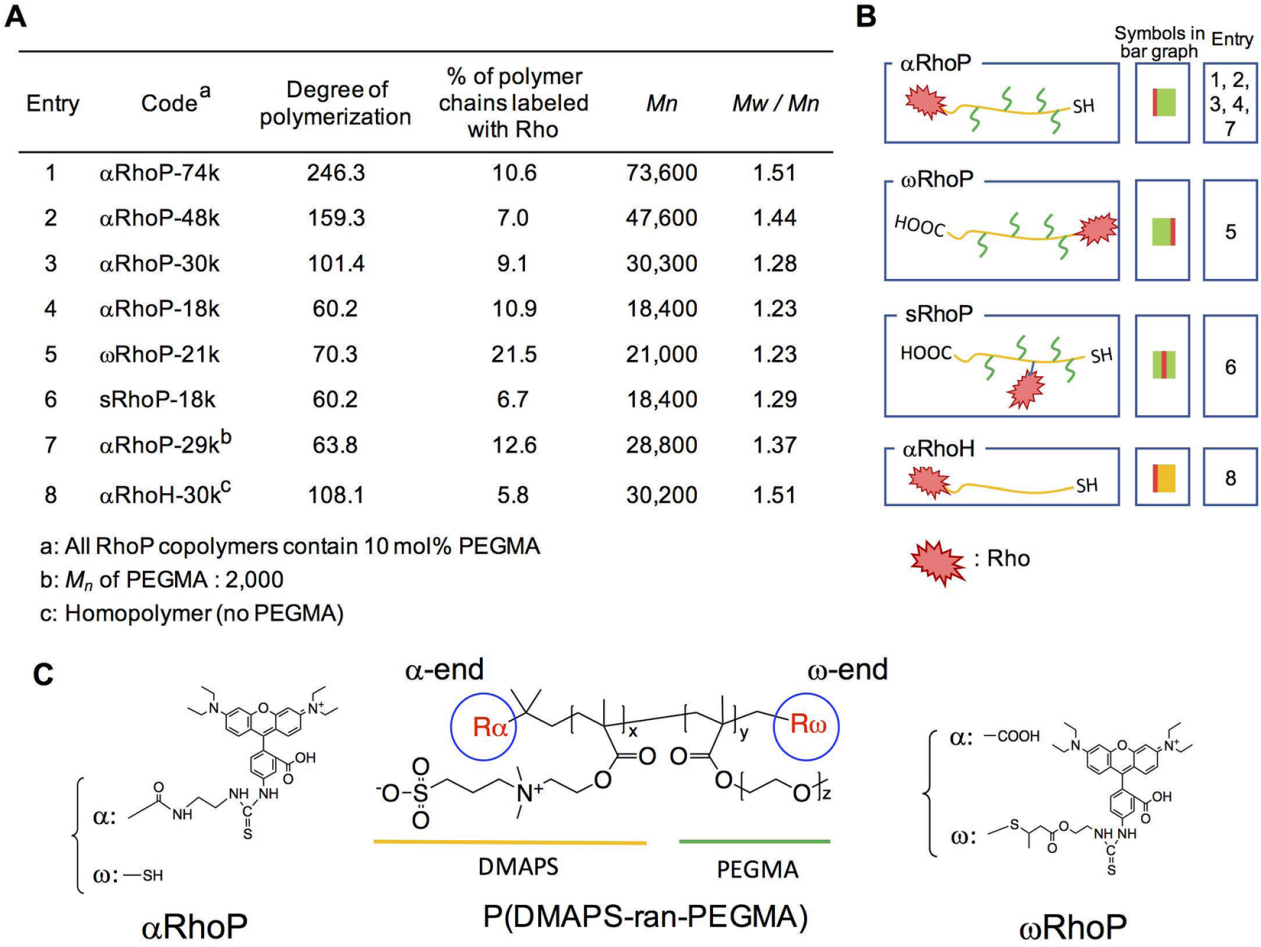
Characteristics and structure of the polymers prepared. (**A**) Table of characteristics for RhoPs. All RhoP polymers contain 10 mol% PEGMA. (**B**) Schematic representation of RhoPs. (**C**) Chemical structure of αRhoP and ωRhoP.

The use of mitochondria-targeted polymeric prodrugs has been explored as an alternative to polymeric nanoparticles. This approach was established by Kopecek *et al*. who selected TPP as targeting group and N-(2-hydroxylpropyl)methacrylamide (HPMA) as drug carriers^24^. We reported recently a new class of mitochondria-targeted polymeric prodrugs able to translocate through the cell membrane and to target the mitochondrion. The polymers, p(DMAPS-ran-PEGMA), contained 3-dimethyl(methacryloyloxyethyl)ammonium propane sulfonate and poly(ethyleneglycol) methacrylate units and a Rho group linked to the α-end of the chain (Fig. 1(C) for the definition of α and ω chain ends)^25^. In saline solutions of physiological pH, the polymer forms loose assemblies, ca 100 nm in diameter, stabilized via ion-pairing between the sulfobetaine groups and via steric stabilization through the PEG chains. This preliminary result encouraged us to undertake a systematic study of the targeting performance of p(DMAPS-ran-PEGMA) samples of different molecular weights and architecture. We prepared copolymers with different amounts of PEG side chains in order to assess the role of the PEG chains in faciliting cell- and mitochondrion-penetration. In addition, the position of the Rho group on the chain, i.e. randomly along the main chain, on the α or on the ω chain end, was controlled through precise chemical manipulation, as a means to modulate mitochondrion targeting and penetration. The results of the study are reported here.

## Results and Discussion

### Precise synthesis of mitochondrion-targeted p(DMPAS-ran-PEGMA) copolymers

The synthesis of the Rho-modified copolymers involved, first, the preparation of unlabeled precursors via RAFT free radical copolymerization of DMAPS and PEGMA and, second, the site-specific linkage of Rho to the copolymer chain. The copolymerization was carried out in a methanol/water mixture rather than the mixed methanol/PBS buffer solution used previously^25^. This solvent led to a better control of the polymerization, resulting in copolymers of narrower molecular weight distribution, particularly in the case of copolymers of molecular weight (*M*_*n*_) less than 30,000 g/mol (See Fig. 1(A)). A terpolymer of DMAPS, PEGMA, and N-methacryloyloxysuccinimide was prepared as precursor to copolymers bearing Rho groups along their main chain. All the precursor copolymers were characterized by ^1^H NMR spectroscopy, to determine the composition of the copolymers, UV-Visible absorption spectroscopy to monitor the absorption band (λ_max_ = 310 nm), ascribed to the trithiocarbonate group, and by gel permeation chromatography for molecular weight determination. (Figures S1 to S3). Most experiments described in this manuscript were performed using a single copolymer sample: p(DMAPS-ran-PEGMA), which contains 10 mol % PEGMA (*Mn* = 500 g/mol). The polymer composition is different from that of the sample used in our previous study (2.5 mol% PEGMA, *Mn* = 2,000 g/mol). However, the weight% of PEGMA is the same in both samples. We changed the PEGMA length and content per monomer unit, in order to ensure that copolymers labeled with Rho remain water-soluble. Consequently, all copolymers, except the copolymer of highest molecular weight (*Mn* = 74,000 g/mol, Entry 1 in Fig. 1(A)) assembled into nanospheres. In contrast, the copolymer of *Mn* = 74,000 g/mol formed much larger particles (hydrodynamic diameter: 30.4 nm, polydispersity index: 0.38). By increasing the number of PEGMA chains along the chain, the formation of large nanospheres was prevented.

By virtue of the RAFT polymerization mechanism and the selection of the chain transfer agent, the precursor copolymers have two different end-groups: a carboxylic acid moiety (α terminus) and an isobutyl trithiocarbonate function (ω terminus). The chemical reactivity of the two groups is not the same, which allowed us to prepare specifically either α-Rho copolymers or ω-Rho copolymers. In all cases, we chose reaction conditions suitable to attain a Rho labelling level ranging from 10% to 20% of the total number of polymer chains. Trial experiments indicated that if the labeling level exceeds ~ 30%, the total emission from internalized Rho-labeled polymers was too intense for fluorescent microscopic observation. Moreover, this low Rho content ensures that all labeled polymers are soluble in physiological solutions without signs of aggregation.

To introduce Rho on the α-position, the α-carboxylic acid was converted to an aminoethylamide by treatment with excess ethylene diamine in the presence of an activator. Subsequently, the modified precursor was reacted with rhodamine B isothiocyanate. This sequence placed Rho moieties on the α end-group with an efficiency of ~ 10%. As indicated in Supplementary Figure S4, this treatment also converted the ω-termini to thiols, since trithiocarbonates undergo facile aminolysis in the presence of primary amines, such as ethylene diamine. All reactions were conducted in the presence of trace amounts of a reducing agent to avoid formation of disulfide linkages. To obtain the ω−Rho-p(DMAPS-ran-PEGMA), ω−RhoP, the ω-trithiocarbonate end group of the precursor copolymer was aminolysed with *n*-butylamine, leading to α−CO_2_H-ω−SH-p(DMPAS-ran-PEGMA), α−RhoP. Michael reaction of 2-aminoethyl methacrylate with the ω-thiol group introduced a primary amine end-group that was reacted with Rhodamine B isothiocyanate. This route lead to a series of α−CO_2_H copolymers labeled with Rho on the ω-end with an efficiency of ~ 20% (Fig. 1(A)).

A copolymer bearing Rho along the main chain was obtained by treatment of p(DMAPS-ran-PEGMA-ran-SuMA) with ethylene diamine, which introduced ~ 3 mol % aminoethyl fragments or ~1.8 ethylenediamine group per polymer chain. Simultaneously, the ω-trithiocarbonate group was converted to a thiol. Reaction of Rhodamine B isothiocyanate with the pendant aminoethyl groups led to the desired labeled polymer (sRhoP-18k). On the basis of the polymer absorbance at 552 nm, we estimate that ~ 7% of the chains are labeled with one Rho along the chain.

### Cellular internalization and localization of Rhodamine-labeled p(DMPAS-ran-PEGMA) samples

**Figure 2:**
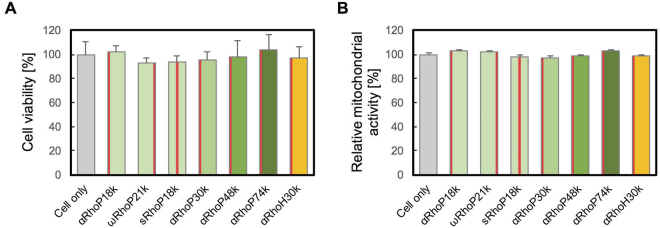
(**A**) Viability of HeLa cells treated with RhoPs (1.0 mg/mL). After addition of each polymer, cells were incubated at 37 °C, 5% CO_2_ for 24 h. The number of cells was evaluated by the Trypan blue assay. (n = 4) (**B**) Relative mitochondrial activity of HeLa cells 24 h upon RhoPs addition. The mitochondrial activity was evaluated by the MTT assay. (n = 4) The red column in each bar of the graph indicates the position of Rho on the polymer. Left: αRhoP, Middle: sRhoP, Right: ωRhoP.

The viability of human cervical carcinoma (HeLa) cells treated with Rho-labeled p(DMPAS-ran-PEGMA)s, RhoPs, containing 10 mol % PEGMA ranging in molecular weight (*M*_*n*_) from 18,000 to 74,000 g/mol labeled with Rho groups either randomly along the chain or at one end group was tested by the Trypan blue assay. The tests indicated that the copolymers (up to 1.0 mg/L, 24 h) do not inhibit the viability of HeLa cells (Fig. 2(A)). Similarly, the mitochondria activity was not affected by the presence of these copolymers (Fig. 2(B)).

**Figure 3:**
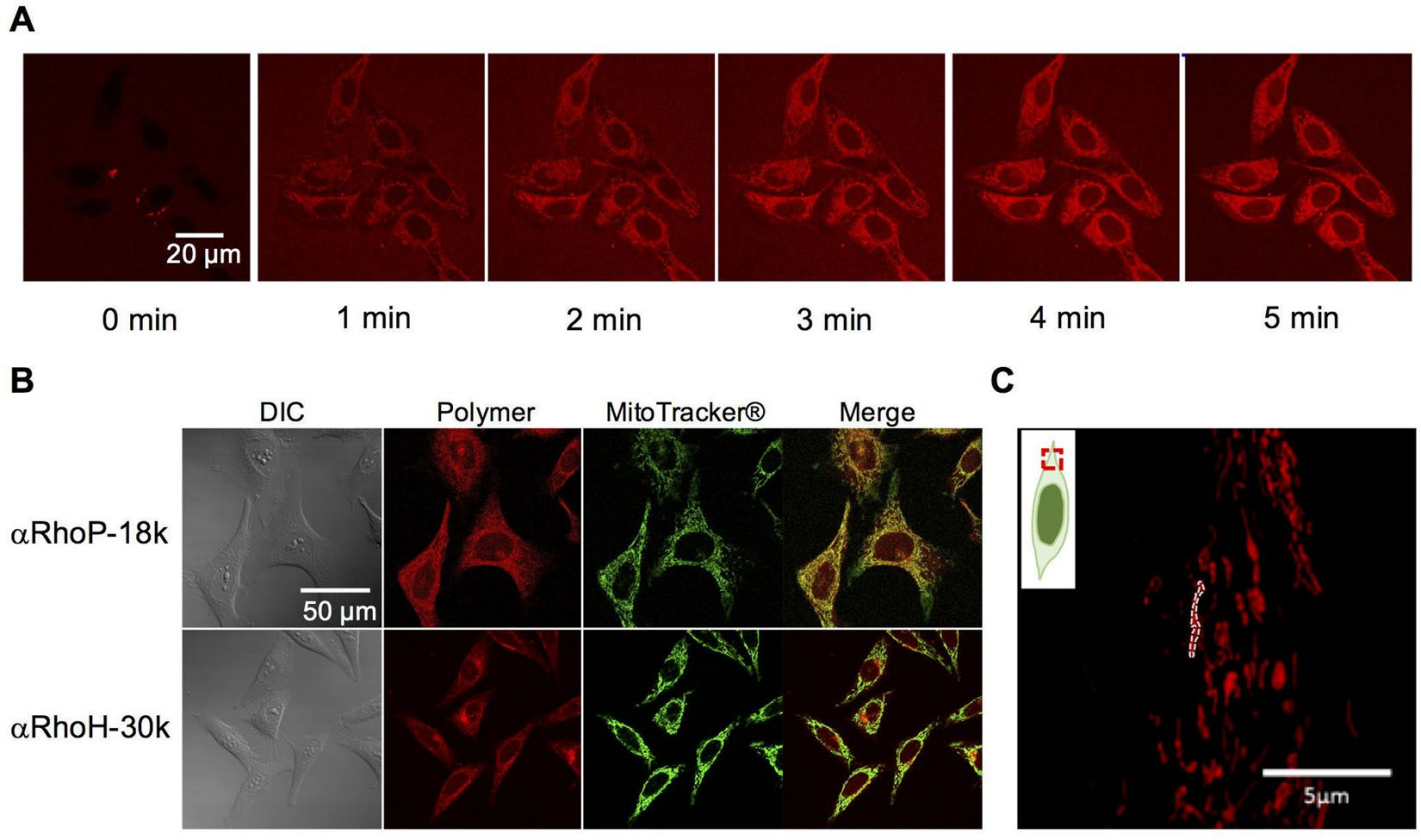
(**A**) Micrographs of HeLa cells at various times post treatment with αRhoP-18k. αRhoP-18k (1.0 mg/mL) was added in the presence of serum. Micrographs were recorded from 0 to 5 min. (**B**) Colocalization of αRhoP (Red) and MitoTracker® (Green) in HeLa cells. After 1 h treatment of αRhoP-18k (upper) and αRhoH-30k (lower) in the presence of serum, MitoTracker was added to the cells. (**C**) Super resolution fluorescence micrograph of αRhoP-18k added to HeLa cell. The white dotted line outlines a mitochondrion. The inset gives a schematic representation of the part of the cell observed.

The copolymers are taken up rapidly by live cells. They localize throughout the cytosol, as shown by the series of time-dependant fluorescence micrographs of cells upon treatment with αRhoP-18k in Fig. 3(A). The cytosol of the cells becomes intensely stained within a few minutes of the injection of the copolymer solution in serum-containing buffer. Such rapid internalization is usually taken as an indication that the mechanism of cellular entry involves translocation through the cell membrane, a point investigated in detail in the next section. Cells treated with the copolymers for 1 h were post-stained with Mitotracker™Green, a known mitochondrion-specific stain. In Fig. 3(B), we present fluorescence micrographs of Hela cells incubated with αRhoP-18k or with αRhoH-30k (red emission) and post-stained with Mitotracker™Green (green emission). Merged micrographs, also shown in Fig. 3(B) confirm the colocalization of the polymers and Mitotracker™Green. Similar results were obtained for all copolymers, independently of their molar mass, the length of the PEG chain, and the position of Rho on the polymer chain (Supplementary Figure S5). The mitochondrial localization of the αRhoP-18k was ascertained further by super-resolution fluorescence microscopy (Fig. 3(C)). The cristae structure can be distinguished in the intensely fluorescent domains within mitochondria, which indicates that the copolymers crossed the inner membrane. The time-lapse video recorded via super resolution microscopy (Supplementary Video S1) revealed the active mobility of mitochondria inferred from the results of the microchondrial activity biochemical assay reported in Fig. 2(B).

### Cellular uptake of Rho-labeled p(DMPAS-ran-PEGMA) samples of different architecture

**Figure 4:**
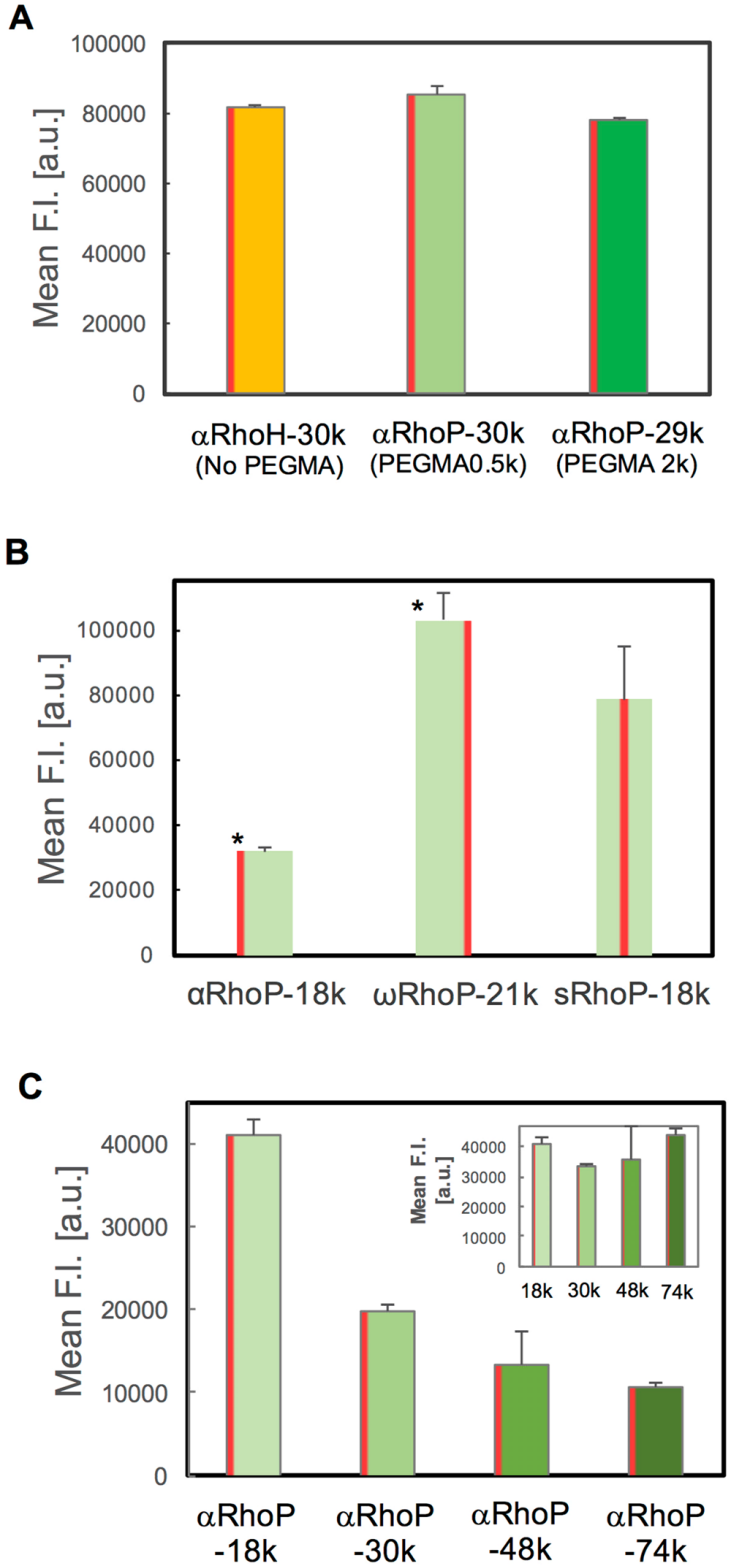
Fluorescence intensity of RhoP-treated HeLa cells. Each polymer was added to HeLa cells and incubated for 1 h. The fluorescent intensity was evaluated by flow cytometry and analyzed for 5,000 cells. For the analysis, the fluorescence intensity values were normalized based on the Rho substitution degree of each polymer. [RhoP] = 1.0 mg/mL (**A**) Effect of the PEG chain length. The substitution degree of PEGMA was approximately the same in all polymers; the molecular weight of PEGMA was 2,000 g/mol (left) and 500 g/mol (right). (**B**) Effect of the position of Rho. The statistical significance of the difference is indicated by * (*p* < 0.01) vs sRhoP. (**C**) Effect of molecular weight of αRhoPs. (inset) Data plotted as a function of the polymer molar concentration.

First, we evaluated by flow cytometry if the cellular uptake of the α-Rho copolymers is affected by the presence and length of the PEG side chains. Copolymers of similar molar mass (*M*_*n*_ ~30,000 g/mol), one without PEG chains (αRhoH-30k) and two with PEG chains (αRhoP-30k, PEGMA 0.5k and αRhoP29k, PEGMA 2.0k) were incubated with Hela cells for 1 h. As shown in Fig. 4(A), there is no significant difference in cellular uptake among the three copolymers. Next, we assessed how cellular uptake is affected by the point of attachment of Rho to the copolymer chain. The normalized fluorescence intensity increased in the order αRhoP < sRhoP < ωRhoP (Fig. 4(B)). The uptake of ωRhoP-21k was ~ 3.2- and 1.3-times higher than the uptake of αRhoP-18k and sRhoP-18k, respectively. It is surprising that the precise site of Rho attachment affects the level of copolymer internalization, since the Rho groups are coupled to either the α or the ω positions by a thiourea group. An ester group connects the thiourea group to the α chain end. This is not the case for the ω position. Partial hydrolysis of the ester group may account for the higher internalization level of ωRhoP-21k, compared to αRhoP-29k. This point is under current evaluation. Then, the effect of the molecular weight (from 18,000 to 74,000 g/mol) was recorded using the αRhoP series (Fig. 4(C)). The fluorescent intensity of cells treated with the polymers decreases with increasing polymer molecular weight. The difference vanishes if one takes into consideration that since the amount of polymer added to cells is kept constant, the number of chains added to a set number of cells decrease with molecular weight. This is observed in the inset of Fig. 4(C), where the uptake data are represented in terms of the molar concentration of monomer units. These results point to the fact that all αRhoP described here have identical membrane translocation ability, independently of the polymer molar mass. The relationship between cellular uptake and polymer concentration was evaluated in the case of αRhoP. There is a first-order correlation between [αRhoP] and cellular uptake (Supplementary Figure S7(B)), which implies that membrane translocation occurs, independently of the level of association of the copolymers in aqueous media (large nanospheres vs loosely-bound chains. In our previous study, cell uptake measurements with copolymer solutions below and above the concentration of nanospheres formation led to the same conclusion. This behavior might be due to the weakness of the association of copolymer chains.

**Figure 5:**
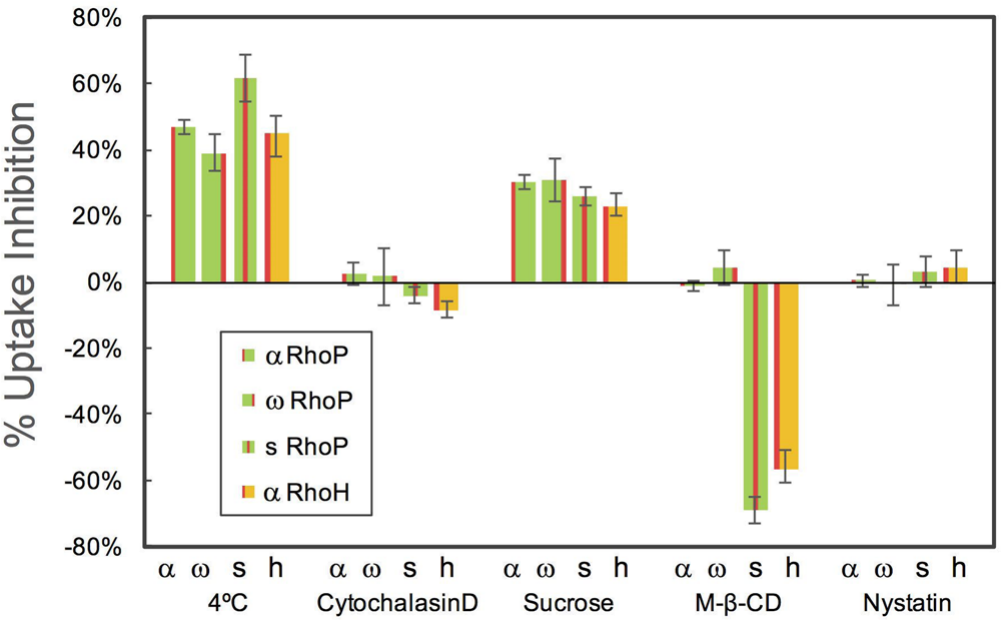
Effect of endocytosis inhibitors on the uptake of RhoPs by HeLa cells. The fluorescence intensity of HeLa cells was evaluated by flow cytometry. The 100% of Mean F.I. are the values of each RhoP in the absence of inhibitor incubated at 37 °C for 1 h. α: αRhoP-18k, ω: ωRhoP-21k, s: sRhoP-18k and h: αRhoH-30k. Cytochalasin D: 10 µM, Sucrose: 0.45 M, MβCD: 10 mM, Nystatin 27 µM.

Further insight on the mechanism responsible for the rapid internalization of αRhoP-18k noted above was gained through studies of the effect of a change in temperature and the presence of pharmacological inhibitors on the efficiency of the uptake of αRhoH-30k and the labeled copolymers αRhoP-18k, ωRhoP-21k, and sRhoP-18k. The results, quantified in terms of the percent uptake inhibition resulting from each treatment are summarized in Fig. 5. The uptake of ωRhoP-21k at 4 °C was inhibited by less than 40%, compared to their uptake by cells kept at 37 °C. It reached 60% in the case of sRhoP-18k. Cooling cells to 4 °C inhibits energy-dependent mechanisms, such as endocytosis. When endocytosis is the exclusive upake mechanism, the uptake inhibition reaches values of ~ 90%, or higher, which is not the case here. Our observation implies that the internalization of the polymers involves several mechanisms. The rapidity of the internalization and the mitochondria localization are strong indicators that translocation occurs; the temperature effect signals that endocytosis is also effective to some extent.

The copolymers uptake was affected by the presence of sucrose and methyl β-cyclodextrin (MβCD), but in opposite directions. Sucrose inhibits the uptake of all RhoPs to some extent. Uptake decreases as the sucrose concentration increases, reaching values ~ 20 and 30% for [sucrose] = 0.3 M and 0.45 M, respectively. Sucrose is known to shut down clathrin-mediated endocytosis. Since neither cytochalasin, an inhibitor of phagocytosis and macropinocytosis, nor nystatin, an inhibitor of caveolin-mediated endocytosis, affect the entry of the copolymers, the overall influence of temperature and the three inhibitors implies that clathrin-mediated endocytosis contributes to the uptake the polymers, albeit to a limited extent. Surprisingly, MβCD enhanced the uptake of sRhoP and αRhoH by as much as 70%, but it did not affect the entry of αRhoP and ωRhoP. MβCD is known to extract cholesterol from the cell membrane, thereby inhibiting caveolae-mediated endocytosis^26^ and lipid raft formation^27^, while enhancing lateral diffusion of phospholipids^28^ and flip-flop mechanisms^29^.

MβCD-induced uptake enhancement was reported previously in the case of cationic oligopeptides, such as oligoarginines^30–32^. Ionic interactions between the cationic oligoarginine and anionic phospholipids involved in the mechanism of oligoarginine uptake affect the cell membrane potential^33^. The cell membrane potential, which results from the difference in ionic strength within the cell and outside, controls the passive transport of small charged molecules. It also affects the internalization of oligoarginine in a K^+^ concentration dependent mode^32^. We measured the effect of the membrane potential on the uptake of RhoPs. Changing the extracellular K^+^ concentration from 0 to 141 mM by exchange with Na^+^ has no effect on the uptake of RhoPs (Figure S7(A)). Hence, we conclude that coulombic interactions between the phospholipidic headgroups and P(DMAPS-ran-PEGMA) are not a controlling factor of the polymers internalization. This observation corroborates the internalization mechanism we proposed in our earlier study of the uptake of fluorescein labeled p(DMAPS-ran-PEGMA) 30k, (PEGMA2.0k) which was dominated by dipole-dipole interactions between the phosphorylcholine moieties of the cell membrane and the sulfobetaines of the polymers^25^. Although Rhodamine B is cationic, it does not participate actively to the transport of the polymers through the cell membrane, nonetheless it may facilitate it. The presence of the cationic Rho becomes important once the polymers reach the cytosol where it contributes to the preferential localization of the polymers in mitochondria.

To summarize, on the basis of a mechanistic study of the uptake by Hela cells of rhodamine B-labeled p(DMAPS-ran-PEGMA)s of different architecture, we established that the uptake is most efficient when Rho is linked to the ω-terminus of the polymer. This leaves the α-terminus available for further modification, such as conjugation of a drug or an imaging agent. The level of PEG modification should be kept low, in the vicinity of 3 ~ 5 mol% of a PEGMA with *M*_*n*_ = 500 g/mol). It is best to select polymers of low molecular weight. This increases the number of chains per weight unit of polymer added, which is equivalent to a higher number of Rho groups per monomer units. Polymers of *M*_*n*_ < 30,000 g/mol are also preferred from the view point of uptake mechanism^25^. The origin of the MβCD discrimination among copolymers of different architecture merits further investigation, as it may point to the design of RhoP with enhanced membrane translocation ability and contribute to the design of effective nanocarriers for mitochondria bioimaging and drug delivery.

## Methods

### Materials

Water (18.2 MΩ·cm) was deionized with Merck Millipore (Darmstadt, Germany) purification system. 2-(1-Isobutyl)sulfanylthiocarbonylsulfanyl-2-methyl propionic acid was prepared as reported previously^34^. 2,2′-Azobis[2-(2-imidazolin-2-yl) propane] (VA-061) was purchased from Wako Pure Chemical Industries (Tokyo, Japan). The inhibitors, poly(ethylene glycol)methyl ether methacrylate (PEGMA, *M*_*n*_ = 500 and 2,000 g/mol), and all other reagents were purchased from Sigma-Aldrich (St. Louis, MO) and used without further purification.

### Synthesis and characterization of p(DMAPS-*ran*-PEGMA) samples

Several p(DMAPS-*ran*-PEGMA) samples were prepared by reversible addition fragmentation transfer (RAFT) copolymerization of PEGMA and DMAPS following a procedure adapted from a previous publication^25^. Suitable amounts of the two monomers (total concentration: 0.1 mol/L), 2-(1-isobutyl) sulfanylthiocarbonylsulfanyl-2-methyl propionic acid, and VA-061 were dissolved in H_2_O/methanol (2/1, v/v). The mixture was purged with N_2_ for 30 min at room temperature. It was brought to 60 °C to induce polymerization and kept at this temperature for 20 to 40 h. The mixture was cooled to 4 °C to stop the polymerization. The polymerization mixture was purified by dialysis against water for 7 days (MWCO = 3,500 g/mol). The polymer was recovered by freeze-drying. Its composition was determined from the ^1^H NMR spectra of the polymers dissolved in D_2_O containing NaCl (1 M) using an ECA-600 spectrometer (JASCO Co. Tokyo, Japan). The molecular weights of the polymers were determined by gel permeation chromatography (GPC) with a JASCO GPC system equipped with TSKgel G3000PW_XL_ and G4000PW_XL_ columns (Tosoh Co. Tokyo, Japan) eluted with aqueous NaNO_3_ (100 mM) and calibrated with PEG standards.

### Synthesis of α-Rho-ω-SH-p(DMAPS-ran-PEGMA) (αRhoP)

1-[3-(Dimethylamino) propyl]−3-ethyl-calbodiimide (EDC, 1.5 equiv.) was added to a solution of p(DMAPS-*ran*-PEGMA) (20 mg/mL,1.0 equiv) in 0.1 M MES buffer (pH 6.0, 10 mL). The resulting mixture was stirred for 1 h at room temperature. A large excess of ethylenediamine (100 equiv.) was added to the mixture in order to convert the α-carboxylic group to the aminoethyl amide and the ω-trithiocarbonate group to a thiol. The mixture was kept at room temperature for 2 h. It was subjected to dialysis (MWCO = 14,000 g/mol) against water for 5 days. A solution of Rhodamine B isothiocyanate (2.0 equiv.) in dimethylsulfoxide, was added to the dialyzed solution. The reaction mixture was kept for 10 h in the dark at room temperature. Subsequently, it was subjected to dialysis for 7 days. The polymer, α-Rho-ω-SH-p(DMAPS-ran-PEGMA), αRhoP was isolated by freeze-drying. The degree of labeling was estimated by UV-Vis spectroscopy analysis of polymer solutions in PBS using the absorbance at 552 nm for Rho (ε = [98,500 M^−1^ cm^−1^]).

### Synthesis of α-CO_2_H-ω-Rho-p(DMAPS-ran-PEGMA) (ωRhoP-21k)

A solution of p(DMAPS-*ran*-PEGMA) (*M*_*n*_ = 21,000 g/mol, 1.0 equiv.) in 1 M NaCl aqueous solution (20 mg/mL) containing a small amount of tris(2-carboxyethyl)phosphine hydrochloride was treated with n-butylamine (100 equiv.) at room temperature for 2 h. The resulting polymer was purified by dialysis for 7 days against water and isolated by freeze-drying. The recovered polymer (1.0 equiv.) was dissolved in a phosphate buffer saline (PBS) containing a small amount of tris(2-carboxyethyl)phosphine hydrochloride and treated with 2-aminoethyl methacrylate (5.0 equiv) at room temperature over a period of 10 h. The mixture was dialysed for 5 days against water. The polymer (α-CO_2_H-ω-SH- p(DMAPS-*ran*-PEGMA) was recovered by freeze-drying and treated with Rhodamine B isothiocyanate as described above to obtain α-CO_2_H-ω-Rho-p(DMAPS-ran-PEGMA), ωRhoP. The degree of labeling was estimated by UV-Vis spectrometry analysis of polymer solutions in PBS using the absorbance at 552 nm for Rho (ε = [98,500 M^−1^ cm^−1^]).

### Preparation of p(DMAPS-*ran*-PEGMA-*ran-*Rho) (sRhoP-18k)

First, the copolymer p(DMAPS-*ran*-PEGMA-*ran*-N-methacryloyloxysuccinimide) was prepared by the same procedure as p(DMAPS-*ran*-PEGMA) using a polymerization mixture of the three monomers: N-methacryloyloxysuccinimide (SuMA), PEGMA, and DMAPS. The monomers (total concentration: 0.1 mol/L), the chain transfer agent and the initiator were dissolved in H_2_O/methanol (2/1, v/v). The mixture was purged with N_2_ for 30 min, and brought to 60 °C to induce polymerization. The mixture was kept at 60 °C for 20 h. It was cooled to room temperature and purified by dialysis against water for 7 days. The polymer recovered by freeze-drying was dissolved in MES buffer (pH 6.0, 20 mg/mL, 1.0 equiv.) and treated with a large excess of ethylenediamine (100 equiv.) to convert the succinimide moieties to aminoethylamides and the ω-trithiocarbonate groups to thiols. The solution was kept at room temperature for 15 h. The resulting polymer was purified by dialysis (MWCO = 14,000 g/mol) against water. A solution of Rhodamine B isothiocyanate (2.0 equiv.) in dimethylsulfoxide was added to the aqueous polymer solution recovered. The reaction was allowed to proceed for 10 h in the dark at room temperature. The polymer, p(DMAPS-*ran*-PEGMA-*ran-*Rho**)** was purified by dialysis for 7 days and isolated by freeze-drying. The degree of Rho-labelling was determined by UV-Vis absorbance spectroscopy.

### Synthesis of α−Rho-ω−SH-p(DMAPS) (αRhoH-30k)

The polymer was obtained by RAFT polymerization of DMAPS, followed by conjugation of Rho to the α terminus of the resulting polymer. Specifically, DMAPS (838.1 mg, 3.0 mmol), 2-(1-isobutyl) sulfanylthiocarbonylsulfanyl-2-methyl propionic acid (7.6 mg, 0.03 mmol), and VA-061 (2.3 mg, 9.0 µmol) were dissolved in H_2_O/methanol (2/1, v/v) (10 mL). The mixture was purged with N_2_ for 30 min at room temperature and brought to 60 °C. The polymerization was let to proceed at 60 °C for 6 h. The resulting mixture was cooled to room temperature and purified by dialysis against water for 7 days (MWCO = 3,500 g/mol). The product was recovered by freeze-drying and subjected to reaction with Rhodamine B isothiocyanate under the conditions described above leading to p(DMAPS) of *Mn* 30,000 g/mol bearing Rho on the α-position and SH on the ω-position (αRhoH-30k).

### Cell culture

HeLa cells were obtained from the Cell Resource Center for Biomedical Research Institute of Development, Aging and Cancer, Tohoku University. They were cultured in Dulbecco’s modified eagle medium supplemented with 10% (v/v) fetal bovine serum. The cultures were maintained at 37 °C in an atmosphere of humidified 95% air and 5% CO_2_ for 24 h. The Rho-labeled polymers dissolved in PBS were added to the cells in amounts necessary to attain a final concentration of 1.0 mg/mL.

### Cytotoxicity test

HeLa cells were seeded at 5,000 cells/well in 96-well microplates and cultured for 24 h. Polymer solutions were added to HeLa cells in amounts such that with their final concentration ranged from 0.1 to 1.0 mg/mL. Treated cells were incubated for 24 h. The cell viability was evaluated by the trypan blue assay using a Countess®FL II automated cell counter (Thermo Fisher Scientific, Waltham, MA).

### Mitochondrial metabolic activity

HeLa cells were seeded at 50,000 cells per 24-well microplates and cultured for 24 h. Polymer solutions were added to HeLa cells in amounts such that with their final concentration ranged from 0.1 to 1.0 mg/mL. Treated cells were incubated for 24 h. Subsequently, the medium was replaced with serum-free medium containing MTT (3-(4,5-dimethylthiazol-2-yl)−2,5-diphenyltetrazolium bromide; 5 mg/mL). Cells were incubated at 37 °C for 30 min. The medium was removed and replaced with DMSO to lyse the cells and to dissolve the formazan produced. Triplicates from each well were collected and placed in a 96-well plate. The absorbance at 595 nm was measured for each well using a microplate reader (SH-9000, Corona electronic, Ibaraki, Japan).

### Cell imaging by fluorescence microscopy

Cells were seeded at a concentration of 200,000 cells per 35 mm glass-bottomed dish and cultured for 24 h. They were washed with PBS and treated with medium containing 10% FBS. Solutions of the Rho-modified p(DMAPS-ran-PEGMA) samples were added to the culture medium in amounts such that their final concentration was 1 mg/mL. The treated cells were incubated for 1 h at 37 °C. They were washed with PBS twice, retreated with PBS and observed using a confocal laser scanning fluorescent microscope (CLSFM) (LSM 5 Pascal, Carl Zeiss, Germany) upon excitation with a He-Ne laser and detection through a 543 nm filter. To confirm the mitochondrial localization of RhoPs in HeLa cells, the cells were treated with Mito Tracker Green (50 nM) for 30 min prior to the addition of the polymers. Super-resolution microscopy was performed using a Nikon N-SIM system consisting of an Eclipse Ti-E microscope equipped with a 100 × /1.49 NA oil immersion objective and a 561 nm solid-state laser. NIS Elements software was used for image capture and super resolution image reconstruction.

### Flow cytometry

HeLa cells were seeded at 50,000 cells per 24-well microplates and cultured for 24 h. After incubation with Rho-labeled polymers (final concentration: 1.0 mg/mL) for 1 h, the cells were washed twice with cold PBS and trypsinized. They were harvested and washed twice with cold PBS. Flow cytometry was performed on a BD Accuri^TM^ C6 flow cytometer equipped with a 488 nm argon laser. AFL2 bandpass emission (585/40) was employed for the detection of Rho. For each sample, 5,000 events were analyzed (n = 4 in each experiment). In the analysis of the flow cytometry data, we took into consideration the differences in the level of Rho incorporation in the polymers by normalizing the fluorescence intensity accordingly. The Student’s t-test was applied to compare the two samples. Values of *p* < 0.01 were considered as significantly different.

### Inhibition of cellular uptake with various inhibitors

HeLa cells were plated at 50,000 cells/mL in 24-well microplates and precultured for 24 h. In the case of assays performed at 4 °C, a solution of a Rho-labeled polymer in cold PBS was added to the cells in amounts such that the final polymer concentration was 1 mg/mL. Cells were incubated for 1 h at 4 °C. The inhibitors, cytochalasin D (3 µM and 10 µM for 15 min)^35^, methyl- β-cyclodextrin, (MβCD, 5 mM and 10 mM for 45 min)^36^, nystatin (11 µM and 27 µM for 30 min)^37^ and sucrose (300 mM and 450 mM for 45 min) were added to the cells^32^. Rho-labeled polymers were added to the inhibitor treated cells to attain a final concentration of 1.0 mg/mL and incubated for 1 h at 37 °C. The cells were incubated for an additional 1 h. The cells were washed with PBS, trypsinized, harvested, and centrifuged at 5,000 rpm for 2 min. The cells were resuspended in cold PBS and evaluated by flow cytometry using the AFL2 bandpass emission (585/40) to detected Rho.

### Effect of membrane potential on cellular uptake

The effect of the membrane potential on cellular uptake of αRhoP-30k was evaluated using a potassium ion concentration ([K^+^]) gradient between the inside and the outside of HeLa cells. The [K^+^] value outside the cell was adjusted by substituting Na^+^ by K^+^ in PBS to maintains the ionic strength at 149.3 mM. The composition of each buffer is shown in Table S1. HeLa cells were plated at 50,000 cells/mL in 24-well microplates and cultured for 24 h. Thereafter, removal the culture media was removed, the cells were washed with PBS washes, the cells were placed in each buffer containing αRhoP-30k with the final polymer concentration of 1.0 mg/mL. After a 5-min incubation at 37 °C, the cells were washed with PBS, trypsinized, harvested, and centrifuged at 5,000 rpm for 2 min. They were resuspended in cold PBS and analysed by flow cytometry using the AFL2 bandpass emission (585/40) to detected Rho. The potassium Nernst potential (E) in each buffer was calculated using Nernst equation:

$$E=(\mathrm{RT}/F)\,\mathrm{ln}\,({{\rm{K}}}_{{\rm{o}}}{/K}_{{\rm{i}}})$$

where R is the gas constant, T is the temperature, F is Faraday’s constant, K_o_ is the potassium ion concentration of outside the HeLa cells, and K_i_ is the potassium ion concentration inside the HeLa cells.

## Electronic supplementary material

Supplementary Information

Supplementary Video S1
