# Supplementary material for: Fast and effective mitochondrial delivery of ω-Rhodamine-B-polysulfobetaine-PEG copolymers
Source: Sci Rep. 2018 Jan 18;8:1128. doi: 10.1038/s41598-018-19598-2 (PMC5773493; doi:10.1038/s41598-018-19598-2)
Supplement: Supplementary file 1 — Supplementary Information [file 41598_2018_19598_MOESM1_ESM.pdf]

# Supplementary Information

# Fast and effective mitochondrial delivery of ω-Rhodamine-B-polysulfobetaine-PEG copolymers

Nobuyuki Morimoto,*,† Riho Takei,† Masaru Wakamura,† Yoshifumi Oishi,† Masafumi Nakayama,‡ Makoto Suzuki,† Masaya Yamamoto,† Françoise M. Winnik.*,§

† Department of Materials Processing, Graduate School of Engineering, Tohoku University, 6-6-02 Aramaki-aza Aoba, Aoba-ku, Sendai, 980-8579, Japan

‡Frontier Research Institute for Interdisciplinary Sciences (FRIS), Tohoku University, Aramaki aza Aoba 6-3, Aoba-ku, Sendai 980-8578, Japan

§Department of Chemistry and Faculty of Pharmacy, University of Montreal, CP6128 Succursale Center Ville, Montreal, QC, H3C 3J7, Canada

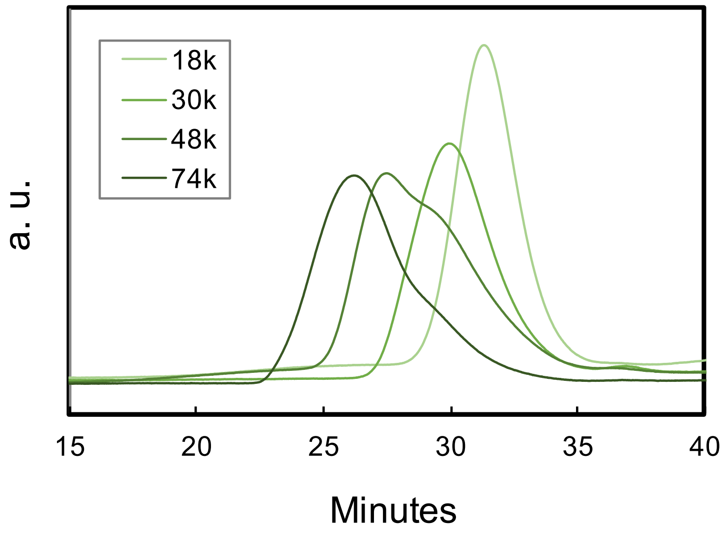


**Supplementary Figure S1.** Gel permeation chromatography (GPC) chromatograms of P(DMAPS-ran-PEGMA). GPC measurements were performed with a JASCO GPC system equipped with TSKgel G3000PW$_{XL}$ and G4000PW$_{XL}$ columns (Tosoh Co. Tokyo, Japan) eluted with aqueous $NaNO_3$ (100 mM). The molecular weights of the polymers were determined by calibration with PEG standards.

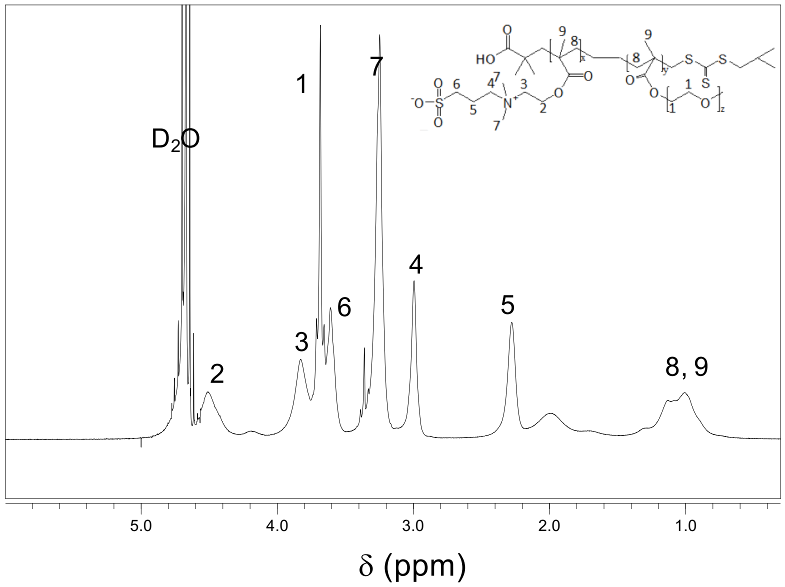


**Supplementary Figure S2.** $^1$H-NMR spectrum of P(DMAPS-ran-PEGMA) in $D_2O$ containing 1 M NaCl.

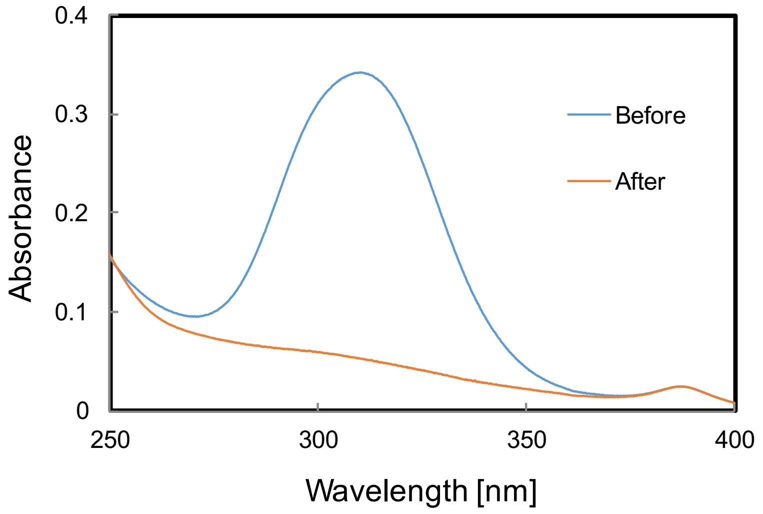


**Supplementary Figure S3. UV-Vis spectra of P(DMAPS-ran-PEGMA) 18k before and after aminolysis reaction.** The aminolysis reaction was applied to P(DMAPS-ran-PEGMA) in MES buffer (pH6.0) by addition of a large excess of *n*-butylamine (100 equiv.) for 2 h at r.t. in order to convert the ω-trithiocarbonate group to a thiol. The absorbance derived from trithiocarbonate group at 310 nm was disappeared by the conversion.

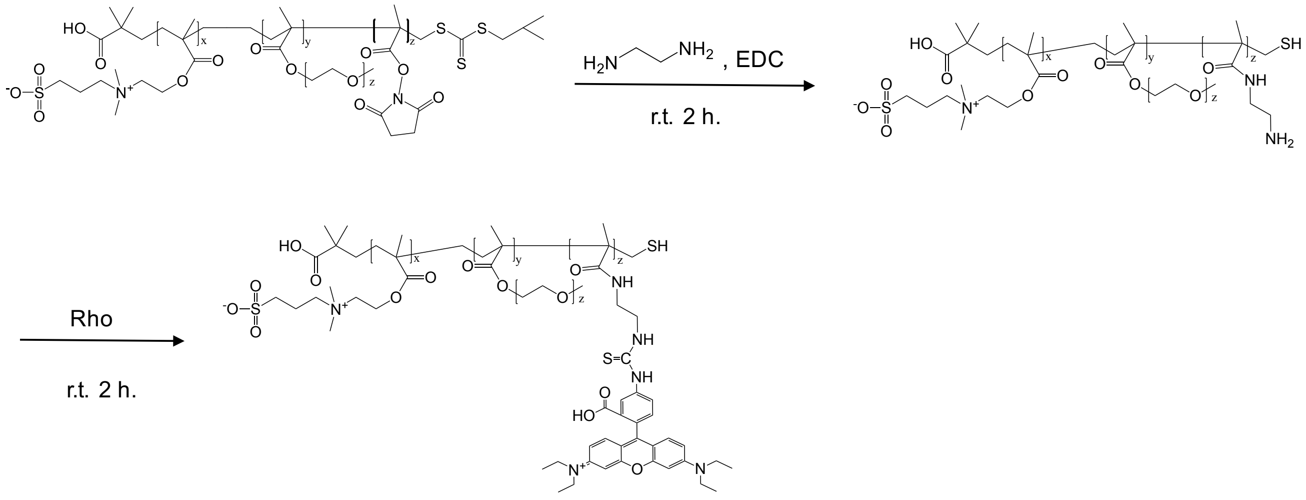


**Supplementary Figure S4. Synthetic scheme of sRhoP**

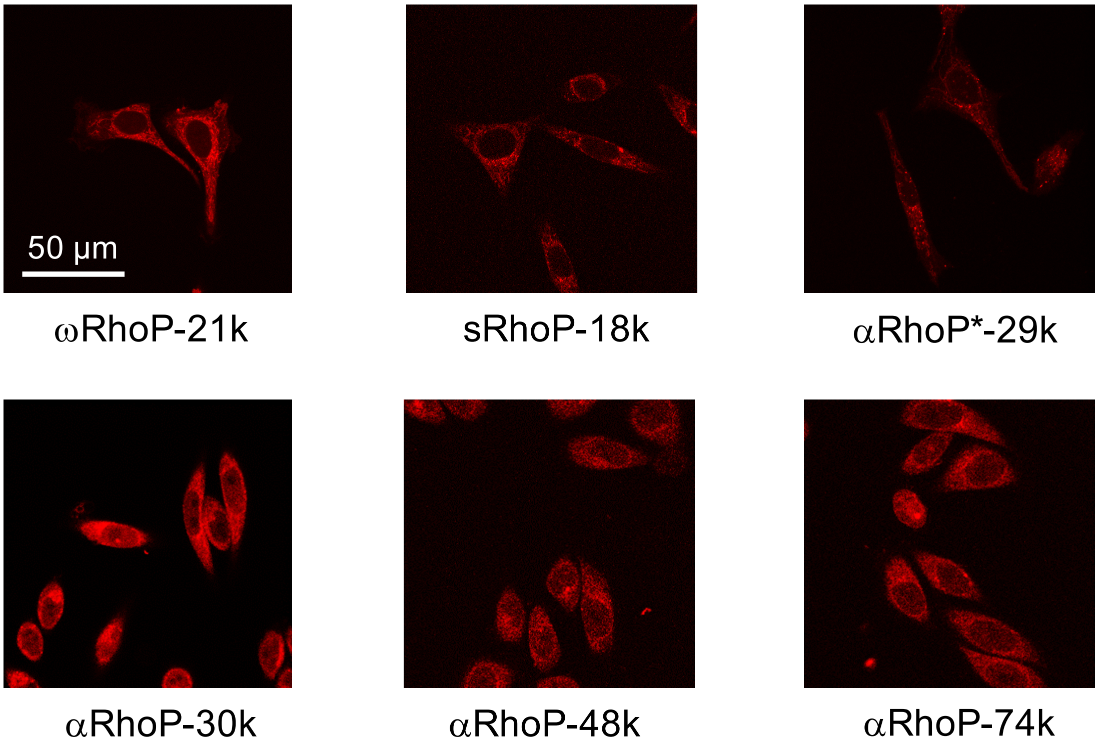


**Supplementary Figure S5. Localization RhoPs in HeLa cells.** The images were obtained by CLSM after 1 h co-incubation with each polymer at 37 ºC, 5% $CO_2$ in the presence of 10 % serum.

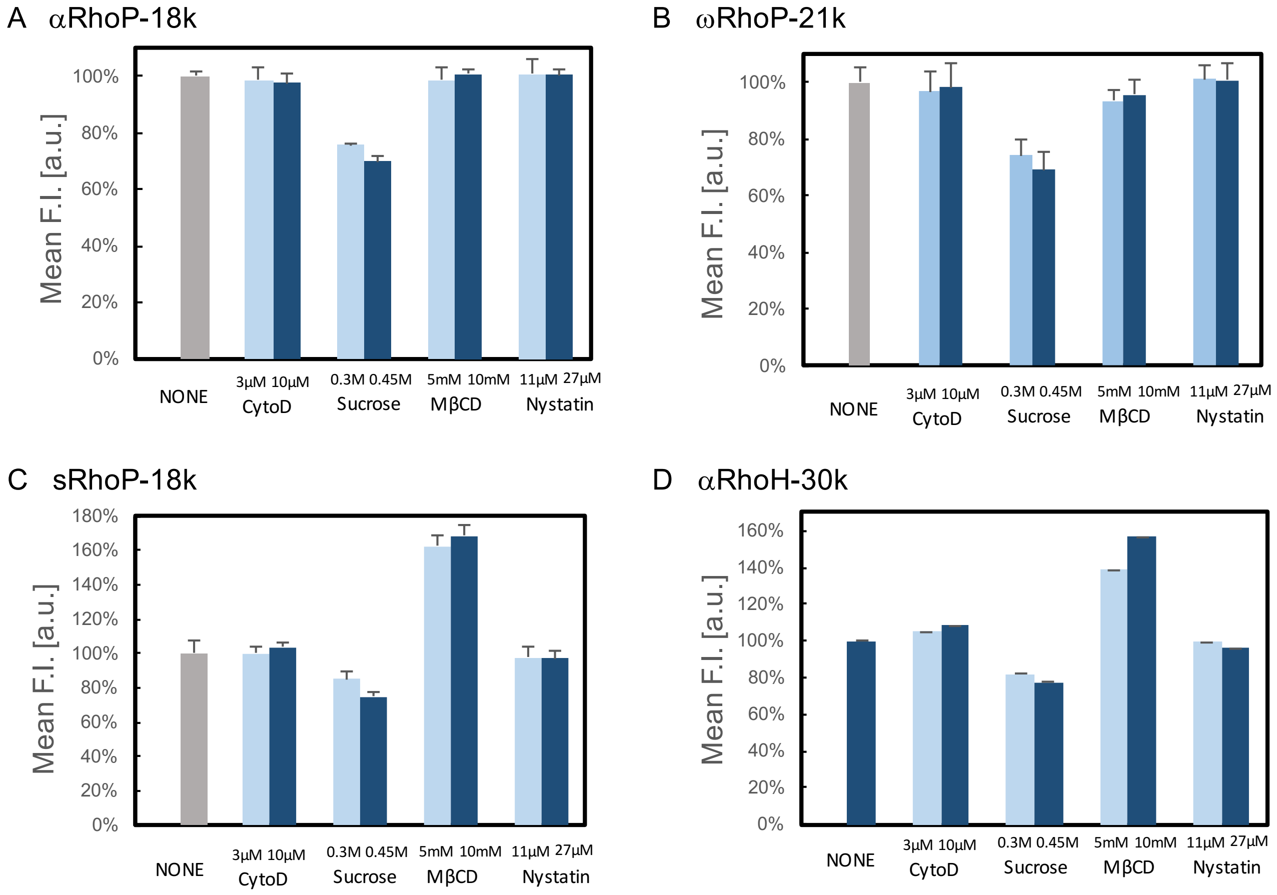


**Supplementary Figure S6. Effects of the concentration of the endocytosis inhibitors on the uptake of (A) αRhoP-18k, (B) ωRhoP-21k, (C) sRhoP-18k and (D) αRhoH-30k.**

The fluorescence intensity of HeLa cells was evaluated by flow cytometry. CytoD: Cytocharacine D, MβCD: methyl-β-cyclodextrin.

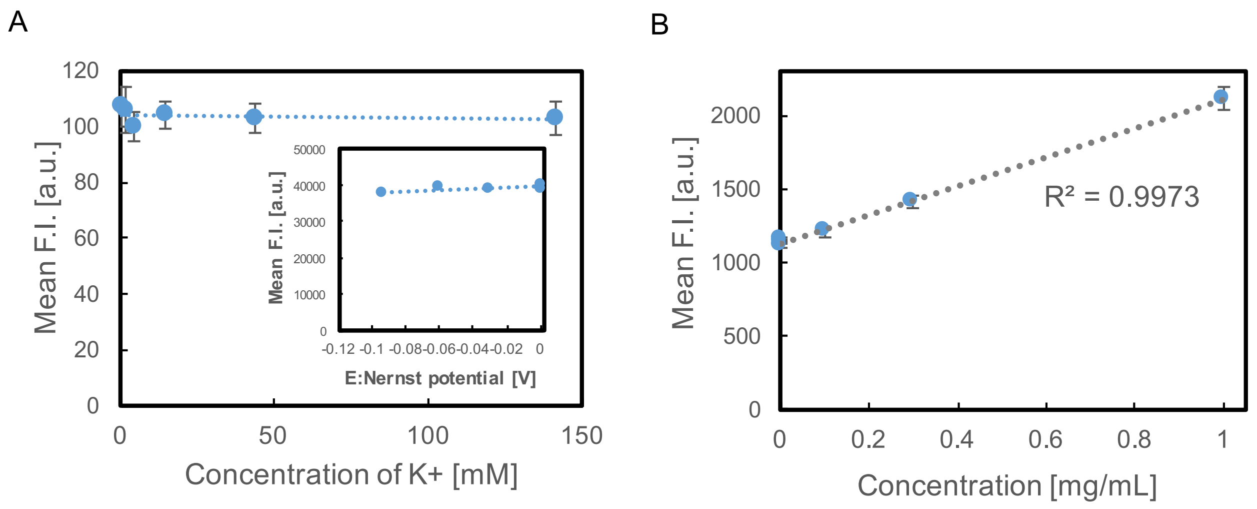


**Supplementary Figure S7.** (A) Fluorescence intensity of αRhoP in HeLa cells as a function of the potassium concentration during polymer uptake. (inset) Plot of the cellular uptake as a function of potassium Nernst potential. (B) Correlation between αRhoP concentration and the amount of cellular uptake. The concentration of αRhoP was ranged from 0.001 mg/mL to 1.0 mg/mL.

**Supplementary Table S1.** Buffer compositions with various potassium concentrations.

| **$[K^+]$ (mM)** | **0** | **1.5** | **4.2** | **14.5** | **43.8** | **141.2** |
|---|---|---|---|---|---|---|
| [NaCl] | 139.7 | 136.7 | 137 | 126.6 | 97.4 | 0 |
| [KCl] | 0 | 0 | 2.7 | 13.1 | 42.3 | 139.7 |
| $[Na_2HPO_4]$ | 9.6 | 8.1 | 8.1 | 8.1 | 8.1 | 8.1 |
| $[KH_2PO_4]$ | 0 | 1.5 | 1.5 | 1.5 | 1.5 | 1.5 |
